# Cytotoxic Lymphocyte-Related Gene Signature in Triple-Negative Breast Cancer

**DOI:** 10.3390/jpm13030457

**Published:** 2023-02-28

**Authors:** Yiqun Han, Jiayu Wang, Binghe Xu

**Affiliations:** Department of Medical Oncology, National Cancer Center/National Clinical Research, Center for Cancer/Cancer Hospital, Chinese Academy of Medical Sciences and Peking Union Medical College, No. 17, Panjiayuan Nanli, Chaoyang District, Beijing 100021, China; han.yiqun@mayo.edu (Y.H.); xubinghebm@163.com (B.X.)

**Keywords:** cytotoxic lymphocytes, breast cancer, tumor microenvironment, cancer immunotherapy, prognosis

## Abstract

To curate the signature genes of cytotoxic lymphocytes (CLs) and explore the heterogeneity based on the CL-related (CLR) gene signature, we analyzed the gene expression of 592 patients with histologically diagnosed triple-negative breast cancer. Based on the 13-gene panel, CLR signatures were curated and associated with the stage of tumor size. Patients in the CLR-low group exhibited the worse overall survival (OS) (median OS, 75.23 months vs. 292.66 months, *p* < 0.0001) and were characterized by the upregulation of the NF-κB, Wnt, and p53 pathways, the positive regulation of angiogenesis, and a higher expression of immune checkpoints including CTLA4, LAG3, CD86, ICOS, ICOSLG, and TNFSF9. In cancer immunotherapy cohorts (GSE157284, GSE35640, IMvigor210), a higher CLR signature score was remarkably associated with greater tumor shrinkage and immune characteristics consisting of higher PD-L1 and neoantigen expression, as well as an inflamed tumor microenvironment. In the pan-cancer atlas, the CLR signature was notably associated with patient survival and revealed a profound heterogeneity across the malignancy types. In sum, the CLR signature is a promising indicator for immune characteristics, tumor shrinkage, and survival outcomes following cancer immunotherapy in addition to the prognostic heterogeneity in the pan-cancer atlas.

## 1. Introduction

Over the past few decades, triple-negative breast cancer (TNBC) has been characterized by aggressive behavior and the least favorable prognosis among breast cancers, for which chemotherapy remains the mainstay of therapeutics for patients with TNBC [1]. The overall survival outcomes of breast cancer have been greatly improved due to the fruitful development and application of novel treatments, yet TNBC remains the leading threat and requires the unmet needs of this distinct subpopulation.

Tumor-infiltrating lymphocytes (TILs) in the tumor tissue have been considered in association with a better prognosis for patients with breast cancer [2]. It has been revealed that TNBC tissue harbors a relatively higher rate of TILs, which has promoted numerous studies focusing on the application of TILs, especially given the in-depth exploration of the tumor microenvironment (TME) and extensive efficacy of immune checkpoint inhibitors [3,4]. Cytotoxic lymphocytes, representative of cytotoxic T lymphocytes (CTLs) and natural killer (NK) cells, play a leading role in the course of the immune response against cancer cells, and their infiltration into the TME is associated with a favorable prognosis [5,6]. Previous studies have extensively proven the linear associations between the increasing presence of TILs and the improved prognosis of patients with TNBC [7,8,9]. Notwithstanding, these studies were conducted based on estimating TILs via hematoxylin–eosin (H&E) staining instead of genomic profiles to give an approximate measurement of the presence of TILs with insufficient attention to classifying TILs and reveling their heterogeneity.

Indeed, previous studies have managed to curate significant genes to estimate the infiltration of CTLs or NK cells. It was suggested that CTL abundance could be assessed using the expression of a five-gene panel [10]. A large gene panel including 116 genes was displayed and enabled the curation of the CTL score for TNBC [11]. Furthermore, a 20-gene panel was introduced and could be utilized to estimate NK cell infiltration in cutaneous melanoma [12]. However, there is no gene constellation curated for CLs as of yet. In addition, TNBC has been considered a highly heterogeneous disease among individuals, in which the interactions between the tumor and the TME have attracted increasing interest [13]. The heterogeneity of the TME, especially the dynamic communication between the tumor and the immune system, could significantly affect cancer development and metastasis. Under these circumstances, the quantification of CLs, as the primary contributor to the immune response against tumors, along with their underlying heterogeneity deserves in-depth illumination, especially under the rapid evolution of cancer immunotherapy.

Herein, we identified the signature genes of CLs and further established the CL-related (CLR) signature based on the gene expression data of patients with TNBC. Utilizing external cohorts, we validated the CLR signature’s associations with clinical outcomes, providing promising values for clinical practice, especially for cancer immunotherapy. We aimed to construct a practical tool serving as a robust biomarker and to facilitate the identification of advantageous populations and the introduction of individual-based therapies.

## 2. Materials and Methods

### 2.1. Patient Selection and Processing

The gene expression profiles of TNBC were searched from the Cancer Genome Atlas (TCGA) [14], the Molecular Taxonomy of Breast Cancer International Consortium (METABRIC) [15], the Gene Expression Omnibus (GEO) [16], ArrayExpress [17], and PubMed databases. The inclusion criteria were as follows: (1) ER and PgR < 1 percentage determined by immunohistochemistry (IHC), and for HER2, either IHC 0 to 1+ or IHC 2+ and fluorescence in situ hybridization negative [18,19]; (2) the corresponding survival outcomes were complete. There were 14 data sets that underwent initial evaluation, and 592 subjects in total were identified in the discovery cohort. The flow diagram of data selection and eligibility is presented in Supplementary Materials Figure S1. Each cohort was assessed individually, and eligible data were evaluated for the batch effect, which was further eliminated by the R *limma* package (version 3.50.1).

### 2.2. Quantification of CL Infiltration

CL abundance was quantifiably estimated using the Microenvironment Cell Populations-counter (MCP-Counter) algorithm [20]. Using this platform, the cytotoxic lymphocyte score, instead of the relative proportion, was computed for each sample, facilitating comparative analyses across the entire population. Associations between cytotoxic lymphocyte abundance and overall survival (OS) were subsequently explored. The effects on survival outcomes of the recapitulative infiltrations estimated by the MCP-Counter platform were evaluated using multivariate regression analysis.

### 2.3. Correlations among CLs and TME Components

The immune score, representing the overall infiltration of immune cells, was appraised using estimate algorithms based on the R *estimate* package (version 2.0.0). The landscape of TME was displayed by xCell, which enumerated the enrichment of 64 immune and stromal components [21]. Accordingly, correlation analysis was carried out to portray the cellular interactions and identify the cell subpopulations in the leading associations with CLs.

### 2.4. Identification of Genes Interacting with CLs

Gene lists were selected following the cell subgroups highly correlated to CLs, of which the signature genes were retrieved from the nCounter Human Immunology Panel (NanoString Technologies). The candidate genes individually underwent a pairwise correlation analysis to assess their degree of interaction with CLs, followed by the successive performance of Cox regression analysis of each gene. Subsequently, the characteristic genes were simultaneously recruited into the Cox proportional hazards model. Only the genes with a *p* value less than 0.05 could be adopted consecutively, and the corresponding genes were acknowledged to not only highly interact with CLs but also exert an independently significant effect on survival. The biological profiles of significant genes were depicted by enrichment analysis conducted by the R *clusterProfiler* package (version 4.2.2).

### 2.5. Curation of the CLR Gene Signature

Based on the significant genes, the CLR signature was introduced and defined as:

$$\overset{n}{\underset{\alpha \;=\;1}{\sum }}G\;\times \;\gamma_{\alpha }-\overset{n}{\underset{\beta \;=\;1}{\sum }}G\;\times \;\gamma_{\beta }$$

where G represents the gene expression and *γ* denotes the hazard ratio (HR) of a specific gene obtained from the multivariate Cox regression analysis. *α* expresses the genes for which the HR was less than 1, while *β* indicates genes with an HR of more than 1. The CLR signature of each patient was finally computed.

### 2.6. Dissection of the CLR Signature-Based Heterogeneity

The CLR signature-based heterogeneity was investigated on the basis of signaling pathways and biological processes pertaining to cancer hallmarks, of which the normalized enrichment score (NES) was curated using single sample gene set enrichment analysis (ssGSEA) using the R *GSVA* package (version 1.42.0). Immune checkpoints were examined to explore the discrepancies in immunologic profiles associated with CLR signatures. The CLR-based heterogeneity was further evaluated across malignancies in addition to the prognostic values of CLR in the TCGA atlas.

### 2.7. Relation between CLR Signature and Cancer Immunotherapy

For external validation, the following cohorts were adopted: GSE157284, containing 82 TNBC samples; GSE35640, including 65 melanomas treated with MAGE-A3 immunotherapy [22,23], which were obtained from the GEO database; and the IMvigor210 cohort, comprising 348 bladder cancer samples treated with PD-L1 antibody retrieved by the R *IMvigor210CoreBiologies* package (version 1.0.0) [24]. The raw data, platform information, and clinical information were obtained, in which the survival outcomes, clinical response, and immunologic characteristics were recorded.

### 2.8. Statistical Analysis

In this study, the cutoff of the CL score and CLR gene signature value was confirmed using maximally selected rank statistics via the R *survival* package (version 3.3-1). The Kaplan–Meier method with log-rank tests was employed to measure the associations between categorical variables and survival parameters. At the same time, a Cox proportional hazards model was established for continuous variables or covariates. The *p* value for multiple comparisons was corrected using the Benjamini–Hochberg procedure [25]. The statistical tests were two-sided, and a *p* value less than 0.05 was considered significant. All statistical analyses were conducted using R software (version 4.1.3).

## 3. Results

### 3.1. Cytotoxic Lymphocyte Abundance and Survival

**Figure 1:**
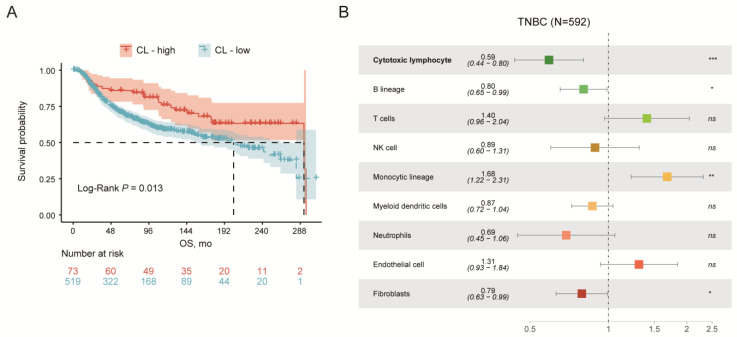
Prognostic values of cytotoxic lymphocytes (CLs) in triple-negative breast cancer (TNBC). (**A**) Prognostic value of the CLR signature-based group in TNBC. (**B**) Prognostic value of cellular compositions in TME of TNBC. ns, *p* > 0.05. ** *p* ≤ 0.01. * *p* ≤ 0.05. *** *p* ≤ 0.001.

Patients with TNBC showed apparently heterogeneous prognoses according to the CL abundance score, in which an ascending CL abundance was associated with superior survival in terms of OS (median OS, 292.67 months vs. 203.53 months, *p* = 0.013) (Figure 1A). After taking into account the majority of TME infiltrations, CLs demonstrated a paramount significance regarding the prognostic effects (*p* < 0.001) (Figure 1B). Taken together, the infiltration of CLs into the TME could be the predominant factor suggestive of the prognosis of patients with TNBC.

### 3.2. Cytotoxic Lymphocytes and TME Compositions

**Figure 2:**
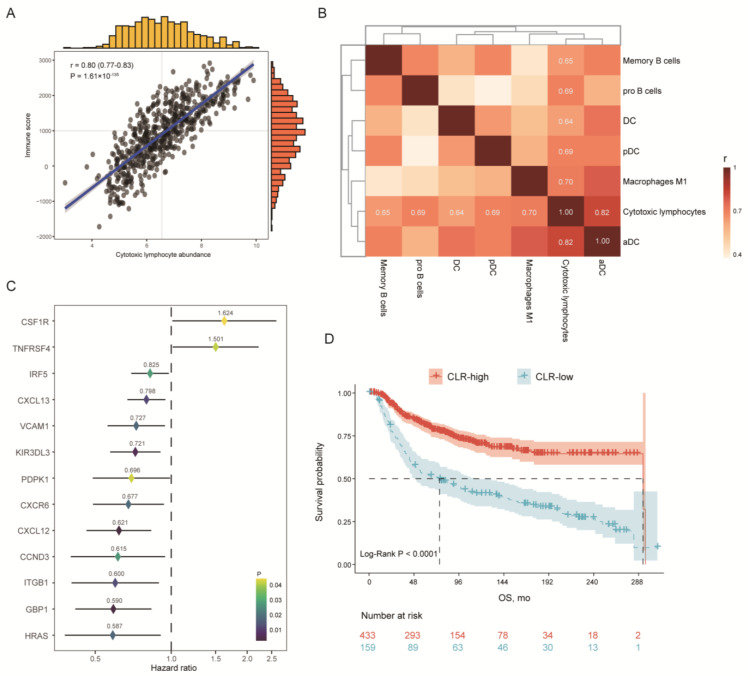
Curation and prognostic value of cytotoxic lymphocyte-related (CLR) signature. (**A**) Correlation between CL abundance and immune score of TME. (**B**) Cellular compositions with the most significant correlation to CLs. (**C**) Signature genes for CLs. (**D**) Prognostic value of CLR signature. CL, cytotoxic lymphocyte. TME, tumor microenvironment. DC, dendritic cells. pDC, plasmacytoid DC. aDC, active DC. OS, overall survival.

The immunologic infiltration of the TME was quantifiably represented by the immune score. It was revealed that CL abundance was strongly correlated to the immune profiles of the TME (r = 0.80, *p* = 1.61 × 10^−135^ (Figure 2A). To visualize the landscape and dissect the cellular heterogeneity in the TME, xCell algorithms were employed (Supplementary Materials: Table S1, Figure S2). Further analysis distinguished that the activated DCs (aDCs) (r = 0.82), M1 macrophages (r = 0.70), plasmacytoid dendritic cells (pDCs) (r = 0.69), pro-B cells (r = 0.69), memory B cells (r = 0.65), and dendritic cells (DCs) (r = 0.64) had close interactions with CLs (Figure 2B).

### 3.3. Cytotoxic Lymphocytes and CLR Signature

According to the findings of the correlation analyses, gene lists were selected associated with the following characteristics and biological processes: antigen processing and presentation, T-cell receptors (TCR), B-cell receptors (BCR), cytokine signaling, and dendritic cell functions. A total of 455 signature genes of these cellular subgroups were collected from the NanoString platform; 248 genes included in our meta-cohort intersected with the selected genes from the nCounter Human Immunology Panel and were finally identified. Based on the enrichment analysis, these shared genes were suggestive of mapping into the immune processes consisting of the cytokine-mediated signaling pathway, leukocyte migration, T-cell activation, leukocyte proliferation, and positive regulation of cytokine production (Supplementary Materials Figure S3).

Subsequently, correlation analysis was conducted between the genes of interest and the abundance of CLs, in which genes were successively assessed regarding their interactive relations with CLs (Supplementary Materials Table S2). Significant genes (110/248) were selected to be analyzed for their prognostic values, and a total of 13 genes were finally identified through the multivariate Cox regression analysis, including *CCND3, CSF1R, CXCL12, CXCL13, CXCR6, GBP1, HRAS, IRF5, ITGB1, KIR3DL3, PDPK1, TNFRSF4*, and *VCAM1* (Supplementary Materials Table S3, Figure 2C). The CLR signature score of each patient was obtained accordingly. Associations between the CLR signature and disease characteristics were explored, which indicated that the CLR signature was negatively associated with the tumor size (Supplementary Materials Figure S4).

**Figure 3:**
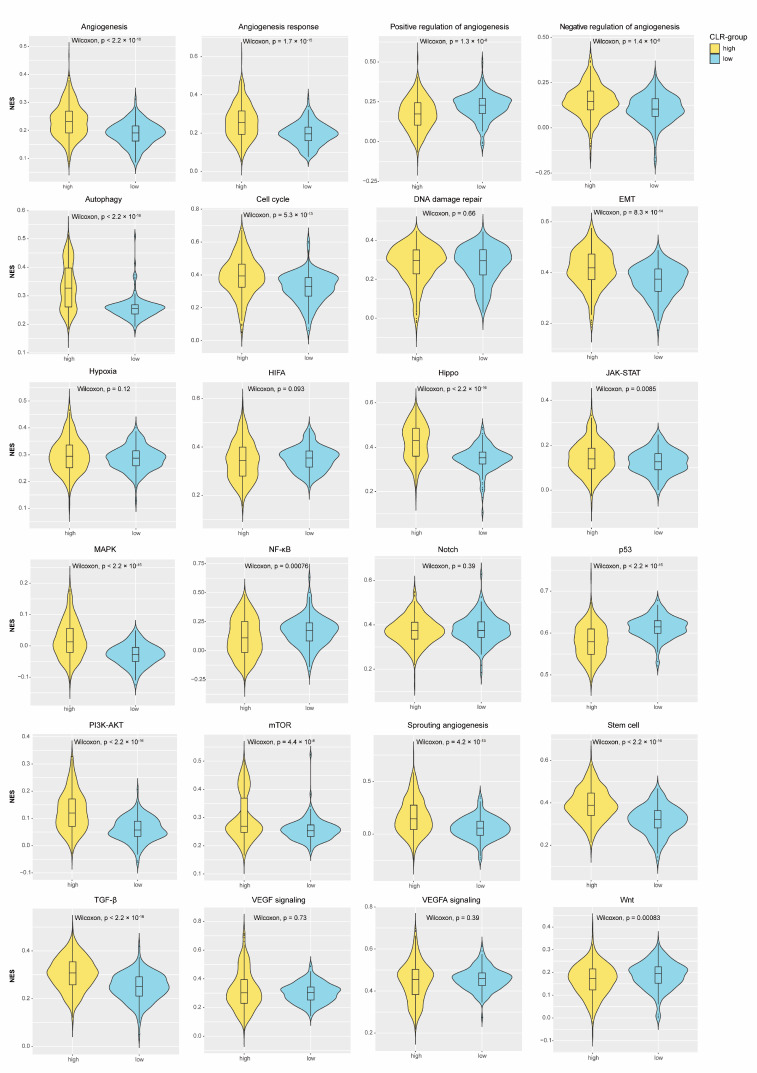
The CLR signature-based heterogeneity of biological pathways.

Based on the CLR signature, patients were divided into two groups with significantly heterogeneous prognoses, and a higher CLR signature was correlated with a better OS (median OS, 292.66 months vs. 75.23 months, *p* < 0.0001) (Figure 2D). The explorative analyses of intrinsic mechanisms, in terms of cancer hallmarks, suggested that the NES values of the CLR-high group were greatly higher in angiogenesis (*p* < 2.2 × 10^−16^, autophagy (*p* < 2.2 × 10^−16^), cell cycle (*p* = 5.3 × 10^−13^), EMT (*p* = 8.3 × 10^−14^), Hippo (*p* < 2.2 × 10^−16^), JAK-STAT (*p* = 0.0085), MAPK (*p* < 2.2 × 10^−16^), PI3K-AKT (*p* < 2.2 × 10^−16^), mTOR (*p* = 4.4 × 10^−8^), and TGF-β signaling pathways (*p* < 2.2 × 10^−16^). By contrast, the CLR-low group was characterized as higher NES values in NF-κB (*p* = 0.00076), p53 (*p* < 2.2 × 10^−16^), and Wnt signaling pathways (*p* = 0.00083). Comparative analyses of the angiogenetic features revealed that the positive regulation of angiogenesis was prominently associated with the CLR-low group (*p* = 1.3 × 10^−8^) (Figure 3).

**Figure 4:**
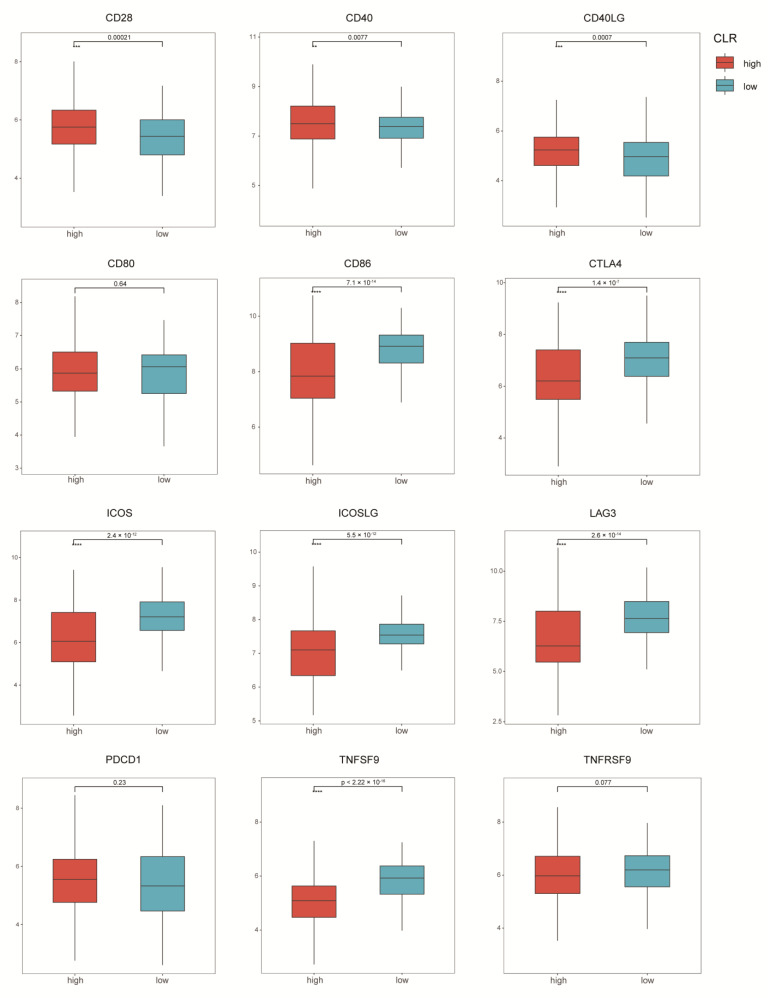
The CLR signature-based heterogeneity of immune checkpoints. ns, *p* > 0.05. ** *p* ≤ 0.01. *** *p* ≤ 0.001. **** *p* ≤ 0.0001.

Concerning the heterogeneity of immune checkpoints, it was demonstrated that the expression of *CD28*, *CD40*, and *CD40LG* was relatively higher in the CLR-high group, while the expression of *CD86, CTLA4, ICOS, ICOSLG, LAG3,* and *TNFSF9* was increased in the CLR-low group when compared to the CLR-high group. No significant difference was detected regarding *PDCD1* (Figure 4).

### 3.4. CLR Signature Associated with Cancer Immunotherapy

**Figure 5:**
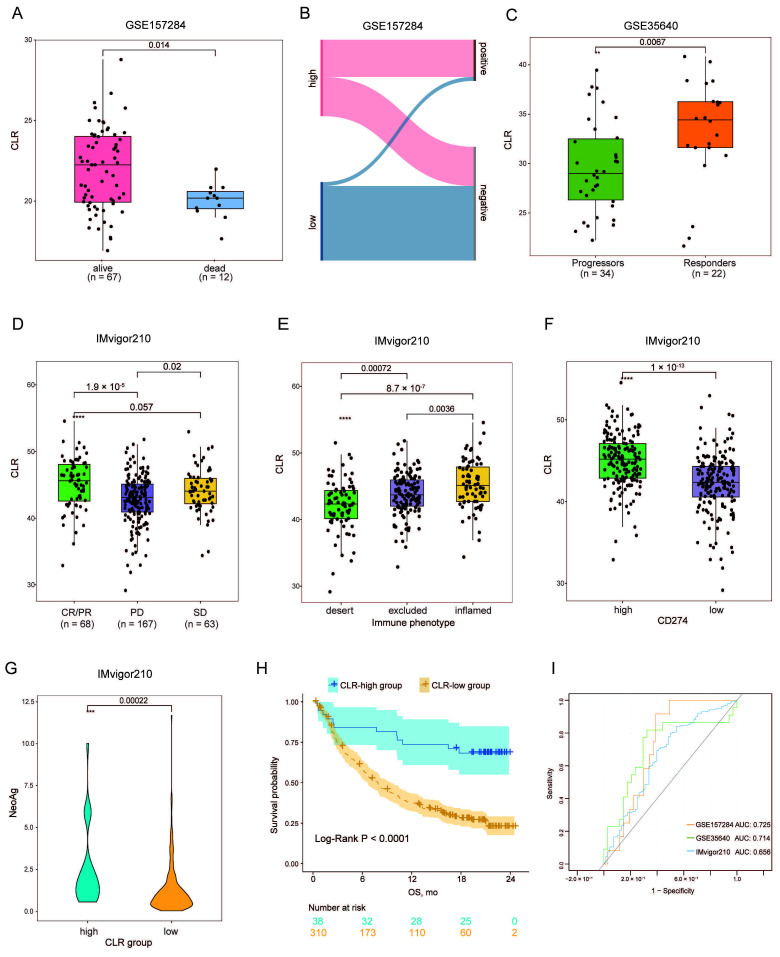
Validation of the CLR signature in cancer immunotherapy cohorts. (**A**) The association between CLR signature and survival status in GSE157284. (**B**) CLR signature versus PD-L1 status in GSE157284. (**C**) The association between CLR signature and clinical response in GSE35640. (**D**) The association between CLR signature and clinical response in IMvigor210. (**E**) The association between CLR signature and tumor microenvironment (TME) subtype in IMvigor210. (**F**) The association between CLR signature and CD274 expression in IMvigor210. (**G**) The association between CLR signature and neoantigen in IMvigor210. (**H**) The association between CLR signature and overall survival (OS) in IMvigor210. (**I**) Predictive value for tumor shrinkage (CR/PR vs. SD/PD) of CLR in GSE157284, GSE35640, and IMvigor210 cohorts. ns, *p* > 0.05. ** *p* ≤ 0.01. *** *p* ≤ 0.001. **** *p* ≤ 0.0001.

To investigate the CLR signature’s performance, external cohorts related to cancer immunotherapy were enrolled, among which only the participants with complete information were adopted into the corresponding analyses. The GSE157284 cohort contained 82 patients with histologically diagnosed TNBC, of which the PD-L1 status was confirmed by the SP142 PD-L1 assay. It was demonstrated that the CLR signature was significantly higher in patients who were still living at follow-up (*p* = 0.014) (Figure 5A). In addition, using the median, a lower CLR signature tended to negatively express PD-L1 (Figure 5B) [22]. In the GSE35640 cohort, we focused on the association between the CLR signature and clinical response. We found that objective responders, defined as those with completely and partially responsive disease, presented relatively higher CLR signatures in comparison to non-responders (*p* = 0.0067) (Figure 5C) [23]. This proportion of the results remained consistent with that of the IMvigor210 data, suggesting that the CLR signature could differentiate the clinical response to PD-L1 antibodies in the bladder cancer population (CR/PR vs. PD, *p* = 1.9 × 10^−5^ (Figure 5D). Regarding the immunologic features, the CLR signature was significantly associated with the immune subtypes, with a declining tendency in the inflamed, excluded, and desert tumors (Figure 5E). In addition, the correlation between the CLR signature and *CD274* expression was of significance, and the CLR signature of the CD274-high group was relatively higher (*p* = 1 × 10^−13^) (Figure 5E). Neoantigens, which are suggestive of tumor genome mutation load, were proven to be higher in the CLR-higher group (*p* = 0.00022) (Figure 5G). Furthermore, the association between the CLR signature and OS was further validated, and patients with a higher CLR score showed a better prognosis than those with a lower value (*p* < 0.0001) (Figure 5H).

### 3.5. CLR Signature in the Pan-Cancer Atlas

**Figure 6:**
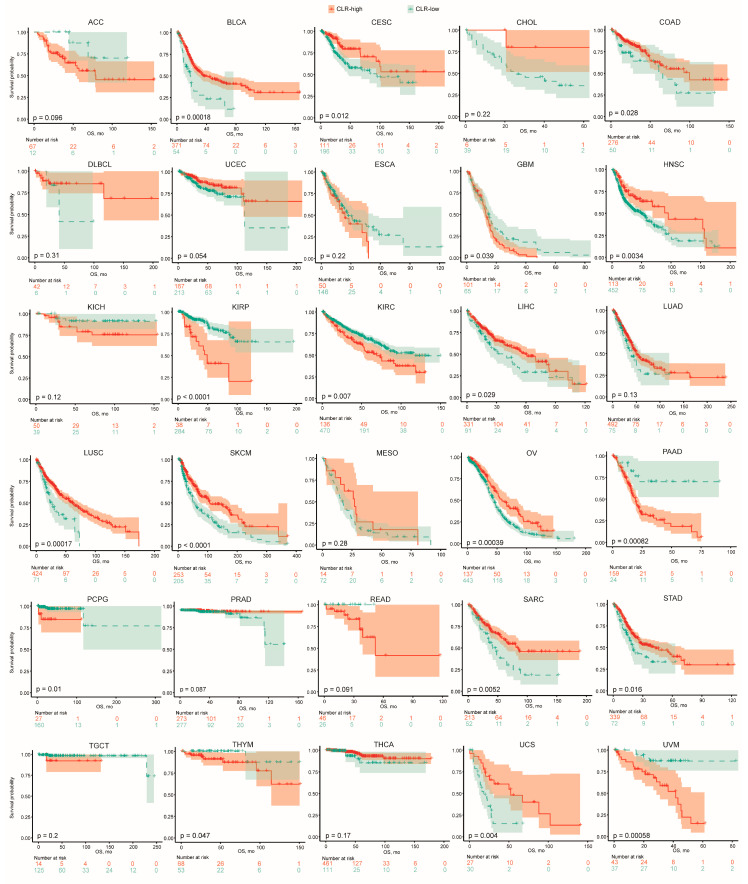
Prognostic values of CLR signature across pan-cancer types. ACC, adrenocortical carcinoma. BLCA, bladder urothelial carcinoma. CESC, cervical squamous cell carcinoma and endocervical carcinoma. CHOL, cholangiocarcinoma. COAD, colon adenocarcinoma. DLBCL, diffuse large B-cell lymphoma. ESCA, esophageal carcinoma. GBM, glioblastoma multiforme. HNSC, head and neck squamous cell carcinoma. KICH, kidney chromophobe. KIRP, kidney renal papillary cell carcinoma. KIRC, kidney renal clear cell carcinoma. LIHC, liver hepatocellular carcinoma. LUAD, lung adenocarcinoma. LUSC, lung squamous cell carcinoma. SKCM, skin cutaneous melanoma. MESO, mesothelioma. OV, ovarian serous cystadenocarcinoma. PAAD, pancreatic ductal carcinoma. PCPG, pheochromocytoma and paraganglioma. PRAD, prostate adenocarcinoma. READ, rectum adenocarcinoma. SARC, sarcoma. STAD, stomach adenocarcinoma. TGCT, testicular germ cell tumor. THCA, thyroid carcinoma. THYM, thymoma. UCS, uterine carcinosarcoma. UVM, uveal carcinoma.

The CLR-based heterogeneity was evaluated across the types of malignancy in the TCGA atlas. It was revealed that squamous cell carcinoma originating from the lung, cervix, and head and neck harbored the highest CLR signature values (Supplementary Materials Figure S5). For the entire population, the highest CLR signature was associated with a superior survival in term of both OS and the progression-free interval (PFI) (Supplementary Materials Figure S6). We then assessed the prognostic effect of the CLR signature regarding the respective malignancies. This kind of effect was demonstrated to be profoundly heterogeneous and varied by cancer type (Figure 6).

## 4. Discussion

Currently, immune checkpoint blockade is being increasing used as a treatment option for patients with TNBC. However, the absence of robust biomarkers to predict response and survival is a notable issue that we cannot shrug off lightly. To our knowledge, this is the first study to define the CL-based signature based on a 13-gene panel as a practical tool. This tool was proven to be reliable and have promising utilization in clinical practice, especially for patients being treated with cancer immunotherapy.

As the primary contributor to prognosis, we focused on the CLs identified from the immune infiltration into the TME of TNBC. To reduce the bias to the utmost extent, we concentrated on the significant genes selected from the associated compositions in the TME, which were significantly correlated to CL abundance and survival. Within the genes of interest, CSF1R participated in the upregulation of PD-L1 in macrophages, and CSF1R inhibition was proven to induce the CTLs infiltration [26]. TNFRSF4 (OX40) is preferentially expressed on regulatory T cells (Tregs) in comparison to effector T cells [27]. Although evidence denoted that OX40 activation could inhibit Tregs and indirectly promote antitumor response, dual roles were recorded, and its ultimate effect remained undetermined [28]. Notably, the research of signature genes was primarily in the preclinical phase. We revealed the predictive values in TNBC, and further studies deserved to be carried out.

A remarkable strength of this study was that we deeply explored the intrinsic mechanisms in association with the novel signature-based prognosis. The nuclear factor-κB (NF-κB) signaling pathway has been considered a critical mediator contributing to proinflammatory reactions and cancer development [29]. In addition, it was proven that the NF-κB family could be recruited to further upregulate PD-L1 expression in TNBC [30]. TNBC could also increase PD-L1 expression, fostering an immunosuppressive TME via the activation of the Wnt signaling pathway, a miscellaneous pathway that participates in the proliferation, metastasis, and therapeutic resistance of breast cancer [31,32,33]. As the most commonly altered gene, *TP53* could interact with the Wnt pathway and promote Wnt-dependent inflammatory reactions to drive tumor metastases [34,35]. A recent study reported that TNBC with chromosomal alternation in which *TP53* is located could reduce CTL infiltration, thus further contributing to the cancer’s aggressiveness [36]. We found that the CLR-low group was characterized by increasing enrichment in this group of pathways. These preclinical findings provide theoretical bases and warrant further studies on the mechanisms of unfavorable prognoses. Additionally, it was shown that the positive regulation of angiogenesis was highly enriched in the CLR-low group, which included the positive regulation of blood vessel branching, sprouting angiogenesis, and vascular wounding healing. Anti-angiogenetic therapy combined with immune checkpoint inhibitors was proven to be promising in clinical application for several malignancies, including TNBC [37]. The functional roles of CLs and CLR signatures should be further validated from the clinical perspective.

The overexpression of inhibitory molecules on cellular components in TME could result in the recruitment of immunosuppressive cell infiltration, which was attributed to a reduction in immunogenicity and cancer immunoediting [38]. Considering the pivotal role in the T cell-mediated immune response, we analyzed the heterogeneity in co-stimulatory and co-inhibitory receptors based on gene expression. CTLA4 was identified as a co-inhibitory receptor to weaken T cell activity; by contrast, CD28, as the co-stimulatory receptor, is involved in this course as an opposing force [39]. We detected a higher CTLA4 expression and a lower CD28 expression in the CLR-low group, indicative of an attenuated immunologic reaction. The co-inhibitory receptor on LAG3 has been widely accepted as a potential target for cancer immunotherapy, and it is also expressed highly in the CLR-low group. These results are consistent with those of CD86 (B7–2), ICOS, the ICOS ligand (ICOSLG), and TNFSF9 (CD137), for which the immune-related characteristics were portrayed by prior studies from different dimensions [40,41,42]. Given that few studies have been focused on the immune checkpoint-related heterogeneity in breast cancer, we provide strong evidence for this kind of prognostic discrepancy.

In the external cohorts receiving cancer immunotherapy, we demonstrated that the CLR signature value was significantly associated with the clinical response to cancer immunotherapy. Responders were revealed to present a relatively higher and more significant CLR value than those who could benefit from cancer immunotherapy. Based on this finding, the CLR signature suggested tumor shrinkage following cancer immunotherapeutic treatment. PD-L1 (B7-H1), encoded by *CD274*, tended to be overexpressed and was considered a definitive biomarker for several cancers treated with anti-PD-1/PD-L1 antibodies [43]. Although this gene was not included in the original dataset, we analyzed the associations between the CLR signature and the PD-L1 status as well as the *CD274* expression in the external cohorts. It was suggested that a high CLR signature value indicated a higher *CD274* expression. Currently, the classification of TME is arousing increasing interest, in which immune abundance is a key indicator for TME subtypes. Several studies have proposed TME classification systems and validated the promising values regarding cancer immunotherapy. The TME has been divided into three subtypes: inflamed, excluded, and desert [44,45,46,47]. This classification was primarily dependent on the CTL activity, and patients with an immune inflamed TME tended to exhibit the best prognosis. Our findings demonstrated a profound association between the TME subtypes and CLR signature according to our findings. Neoantigens were considered the target of the immune response, which could be recognized by T effector cells. An increased immune infiltration was observed correlated to a higher tumor neoantigen burden [10,48,49]. A previous study confirmed the definite correlation between the mutational load and the CL activity. The heterogeneity in antigenic profiles tended to be associated with the response to immune checkpoint blockade [10,50]. This was consistent with our finding that the CL-based signature was significantly correlated to the neoantigens in the cancer immunotherapy cohort.

Previous studies have proven that the presence of TILs in tumors predicts a superior prognosis of patients with colon, lung, cervical, and ovarian cancers as well as melanoma [51,52,53]. However, not all malignancies were covered. Thus, this kind of association is yet to be extensively determined. In this study, we unprecedently assessed the prognostic value of CL-based signature for the pan-cancer atlas. Concerning the malignancy types, profound heterogeneity was revealed. The results of the aforementioned cancer types remained consistent. It was demonstrated that the CLR gene signature could differentiate the survival of patients with bladder cancer, liver hepatocellular carcinoma, head and neck squamous cell cancer, sarcoma, stomach carcinoma, and uterine carcinosarcoma. However, an inverse correlation was detected in patients suffering from glioblastoma, renal clear cell carcinoma, renal papillary cell carcinoma, pancreatic ductal carcinoma, and uveal melanoma. Taken together, we provided new evidence regarding the application of the CLR signature in the pan-cancer population. The heterogeneity of CTL has increased over the past decade [54]. Although CLs are able to infiltrate the tumor, chances are that they are in a dysfunctional state and, accordingly, cannot promote the anti-tumor immune response [55]. Under these circumstances, multi-omics disciplinary studies should be incorporated to reveal the heterogeneity in the TME as the ultimate event of immune response, which is not likely to be determined by a comprehensive process instead of assessing gene expression. More effective biomarkers are necessary to curate an increasingly predictive model.

For starters, the retrospective nature of our study could lead to potential bias. Next, a group of clinicopathological information was missing in the public database; for instance, the follow-up period could be variable or insufficient for survival outcomes without a definite record. Lastly, although we have managed to decrease the bias regarding gene selection, chances are that some genes could be neglected. Future experiments are worth carrying out to further validate the CLR-based signature and the underlying mechanisms for prognostic heterogeneity.

In conclusion, this study established a robust gene signature based on the CL infiltration in TNBC, which was associated with tumor size, immune characteristics, response, and survival outcomes following cancer immunotherapy, as well as prognostic heterogeneity in the pan-cancer atlas. Considering there was no current consensus on the generalized biomarkers of immunotherapy for breast cancer, our study provided promising evidence for this undetermined point in the current cancer immunotherapy era.

## Data Availability

The data sets for this study can be found in The Cancer Genome Atlas (TCGA) data set (https://xenabrowser.net/datapages/, accessed on 1 September 2022), Molecular Taxonomy of Breast Cancer International Consortium (METABRIC, http://www.cbioportal.org/, accessed on 1 September 2022), ArrayExpress (https://www.ebi.ac.uk/arrayexpress/, accessed on 1 September 2022), the Gene Expression Omnibus (GEO, https://www.ncbi.nlm.nih.gov/geo/, accessed on 1 September 2022), and the PubMed database (https://pubmed.ncbi.nlm.nih.gov/, accessed on 1 September 2022).

## Supplementary Materials

The following supporting information can be downloaded at: https://www.mdpi.com/article/10.3390/jpm13030457/s1. Figure S1. Flow diagram of patient selection and identification. Figure S2. Correlation among cytotoxic lymphocytes abundance and other cellular constitutions in tumor microenvironment of triple-negative breast cancer. Figure S3. Enrichment analysis of overlapping genes identified in the selected gene set. Figure S4. The association between the CLR signature and disease characteristics. Figure S5. The CLR signature-based heterogeneity across cancer types. Figure S6. The associations between CLR signature and overall survival (A) and progression-free interval (B). Supplementary Materials Table S1. Correlation coefficients among the cellular populations of tumor microenvironment. Supplementary Materials Table S2. Correlation analysis between candidate genes and cytotoxic lymphocyte abundance. Supplementary Materials Table S3. Signature genes for cytotoxic lymphocytes.
