# Supplementary material for: Cytotoxic Lymphocyte-Related Gene Signature in Triple-Negative Breast Cancer
Source: J Pers Med. 2023 Feb 28;13(3):457. doi: 10.3390/jpm13030457 (PMC10054905; doi:10.3390/jpm13030457)

Figure S1. Flow diagram of patient selection and identification.

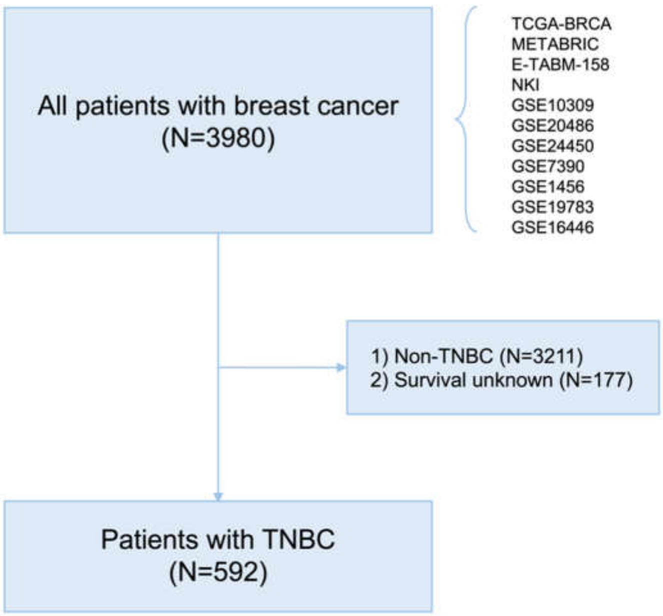

Figure S2. Correlation among cytotoxic lymphocytes abundance and other cellular constitutions in tumor microenvironment of triple-negative breast cancer.

DC, dendritic cells. aDC, active DC. cDC, classical DC. iDC, interstitial DC. CD4 Tem, CD4 T effector memory cells. CD8 Tem, CD8 T effector memory cells. Tregs, regulatory T cells. pDC, plasmacytoid DC. CD8 Tcm, CD8 T central memory cells. MPP, multi-potent progenitor cells. MEP, megakaryocyte-erythroid progenitor cells. NKT, natural killer T cells. CMP, common myeloid progenitor cells. CD4 Tcm, CD4 T central memory cells. MSC, mesenchymal stem cells. Tgd cells, gamma delta T cells. HSC, hematopoietic stem cells. GMP, granulocyte/monocyte progenitor. CLP, common lymphoid progenitor.

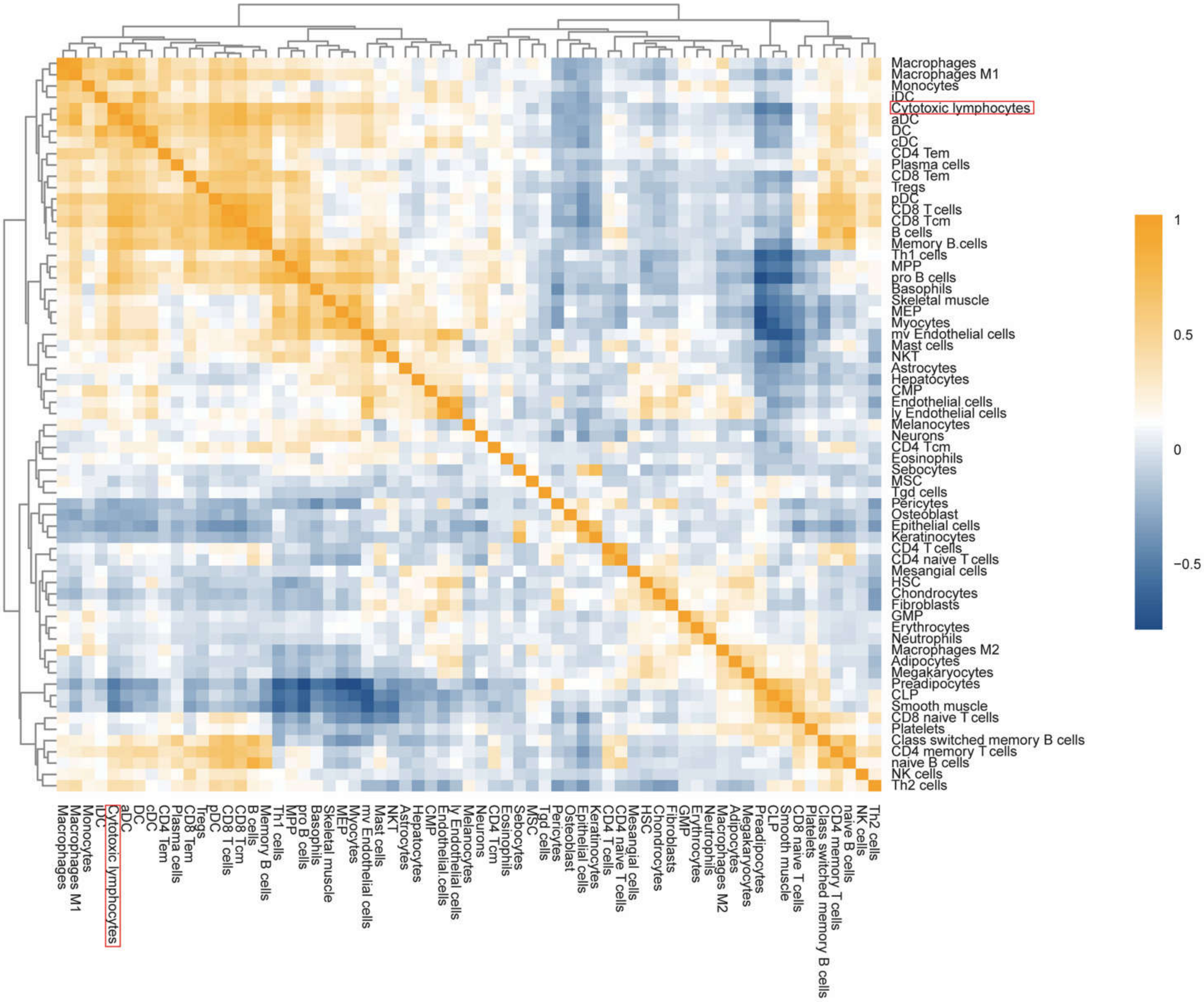

Figure S3. Enrichment analysis of overlapping genes identified in the selected gene set.

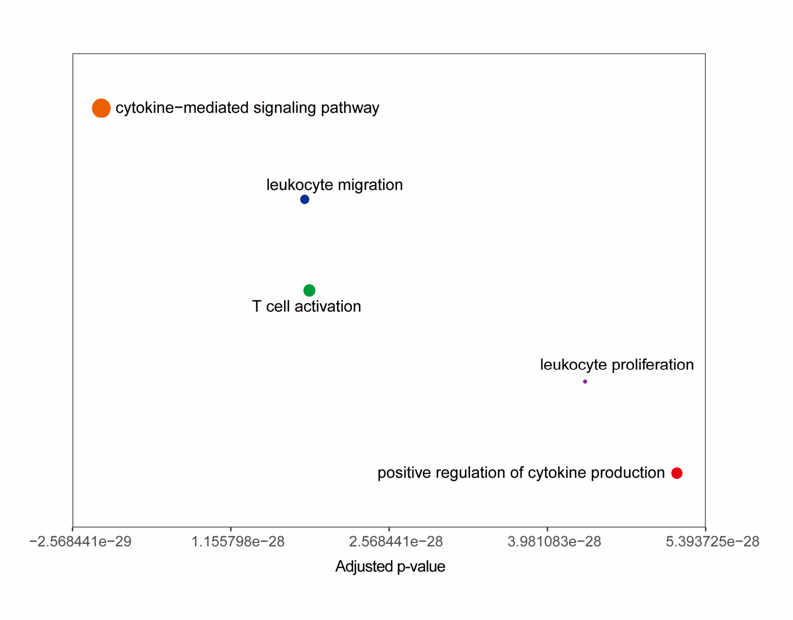

Figure S4. The association between the CLR signature and disease characteristics.
(A) The association between the CLR signature and tumor size stage. (B) The association between the CLR signature and node metastasis status. (C) The association between the CLR signature and distant metastasis status. (D) The association between the CLR signature and AJCC TNM stage.

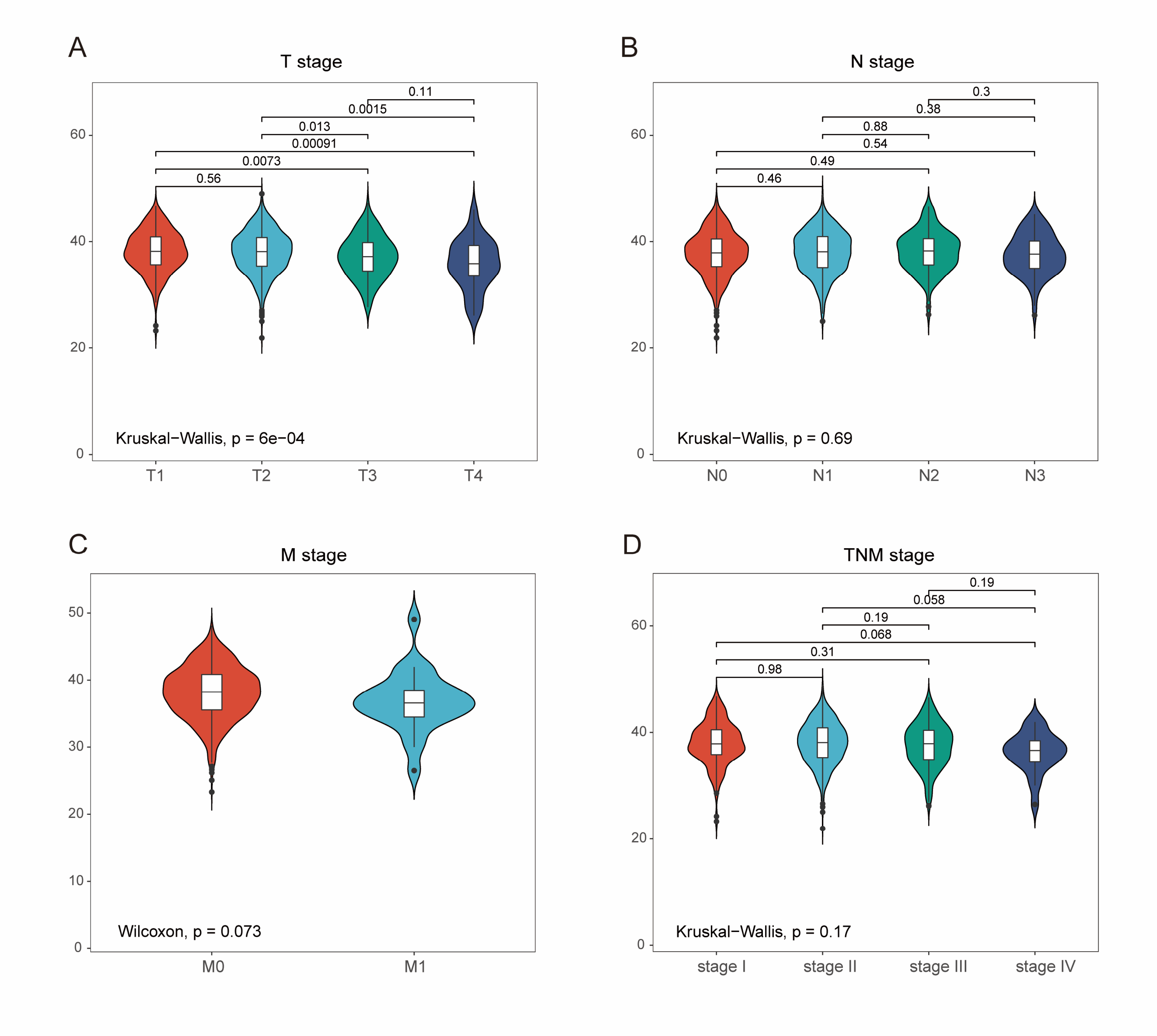

Figure S5. The CLR signature-based heterogeneity across cancer types.

ACC, adrenocortical carcinoma. BLCA, bladder urothelial carcinoma. CESC, cervical squamous cell carcinoma and endocervical carcinoma. CHOL, cholangiocarcinoma. COAD, colon adenocarcinoma. DLBCL, diffuse large B-cell lymphoma. ESCA, esophageal carcinoma. GBM, glioblastoma multiforme. HNSC, head and neck squamous cell carcinoma. KICH, kidney chromophobe. KIRP, kidney renal papillary cell carcinoma. KIRC, kidney renal clear cell carcinoma. LIHC, liver hepatocellular carcinoma. LUAD, lung adenocarcinoma. LUSC, lung squamous cell carcinoma. SKCM, skin cutaneous melanoma. MESO, mesothelioma. OV, ovarian serous cystadenocarcinoma. PAAD, pancreatic ductal carcinoma. PCPG, pheochromocytoma and paraganglioma. PRAD, prostate adenocarcinoma. READ, rectum adenocarcinoma. SARC, sarcoma. STAD, stomach adenocarcinoma. TGCT, testicular germ cell tumor. THCA, thyroid carcinoma. THYM, thymoma. UCS, uterine carcinosarcoma. UVM, uveal carcinoma.

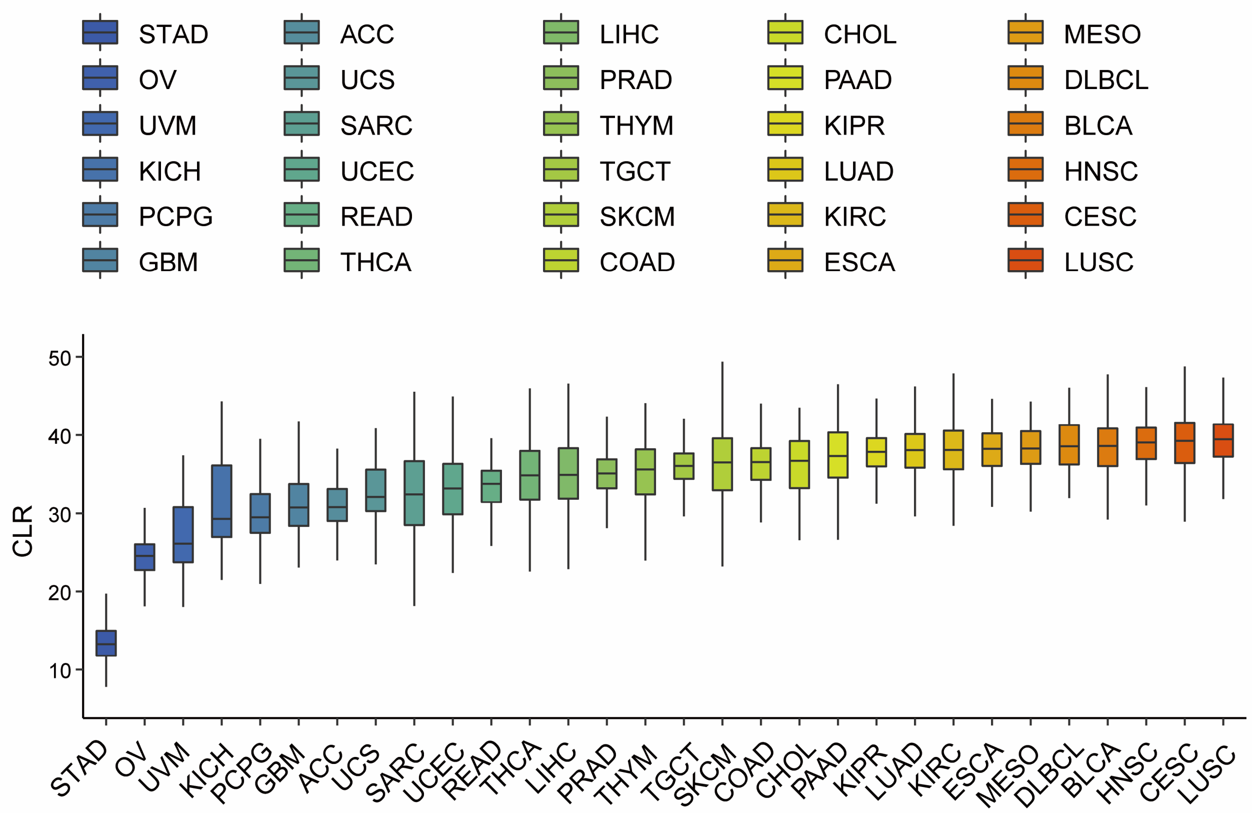

Figure S6. The associations between CLR signature and overall survival (A) and progression-free interval (B).

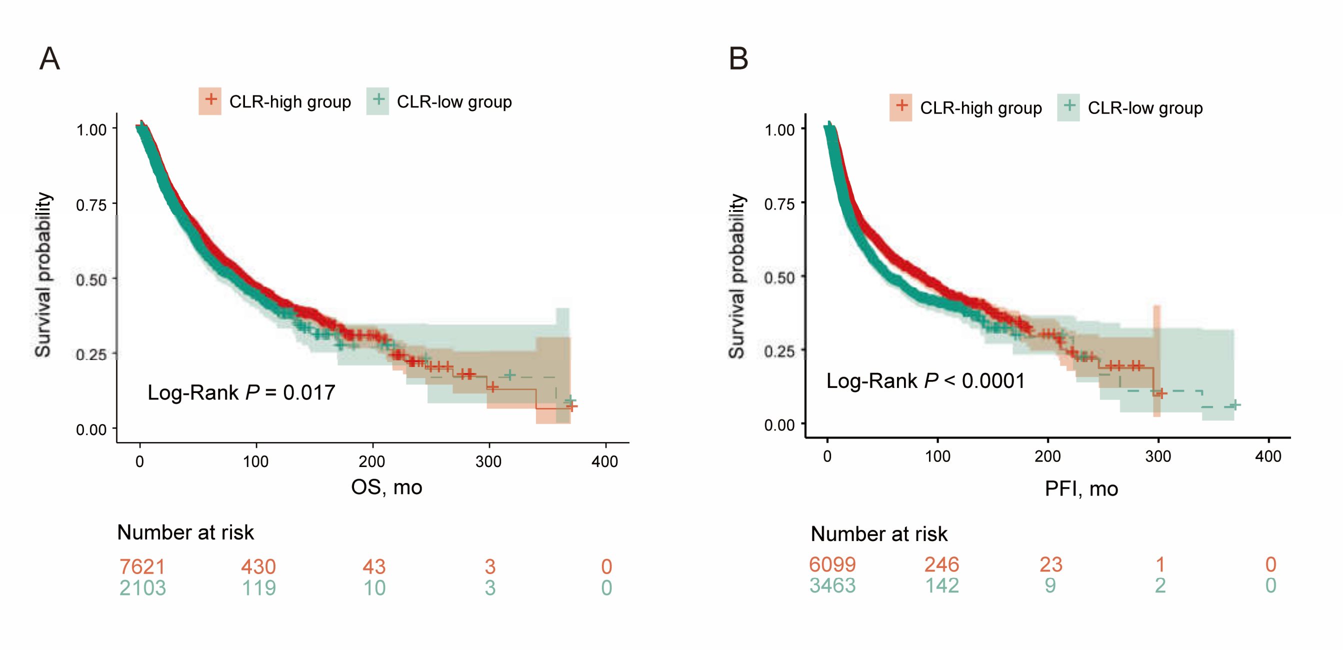

Supplement: Supplementary file 1 [file jpm-13-00457-s001.zip › Supplementary Figures.pdf]
